# Intertwined roles for GDF-15, HMGB1, and MIG/CXCL9 in Pediatric Acute Liver Failure

**DOI:** 10.3389/fsysb.2024.1470000

**Published:** 2024-10-15

**Authors:** Ruben Zamora, Jinling Yin, Derek Barclay, James E. Squires, Yoram Vodovotz

**Affiliations:** ^1^ Department of Surgery, University of Pittsburgh, Pittsburgh, PA, United States; ^2^ Center for Inflammation and Regenerative Modeling, McGowan Institute for Regenerative Medicine, Pittsburgh, PA, United States; ^3^ Pittsburgh Liver Research Center, University of Pittsburgh, Pittsburgh, PA, United States; ^4^ Department of Pediatrics, University of Pittsburgh, Pittsburgh, PA, United States

**Keywords:** inflammation, biomarker, network analysis, serum, systems biology

## Abstract

**Introduction:**

Pediatric Acute Liver Failure (PALF) presents as a rapidly evolving, multifaceted, and devastating clinical syndrome whose precise etiology remains incompletely understood. Consequently, predicting outcomes—whether survival or mortality—and informing liver transplantation decisions in PALF remain challenging. We have previously implicated High-Mobility Group Box 1 (HMGB1) as a central mediator in PALF-associated dynamic inflammation networks that could be recapitulated in acetaminophen (APAP)-treated mouse hepatocytes (HC) *in vitro*. Here, we hypothesized that Growth/Differentiation Factor-15 (GDF-15) is involved along with HMGB1 in PALF.

**Methods:**

28 and 23 inflammatory mediators including HMGB1 and GDF15 were measured in serum samples from PALF patients and cell supernatants from wild-type (C57BL/6) mouse hepatocytes (HC) and from cells from HC-specific HMGB1-null mice (HC-HMGB1^−/−^) exposed to APAP, respectively. Results were analyzed computationally to define statistically significant and potential causal relationships.

**Results:**

Circulating GDF-15 was elevated significantly (*P* < 0.05) in PALF non-survivors as compared to survivors, and together with HMGB1 was identified as a central node in dynamic inflammatory networks in both PALF patients and mouse HC. This analysis also pointed to MIG/CXCL9 as a differential node linking HMGB1 and GDF-15 in survivors but not in non-survivors, and, when combined with *in vitro* studies, suggested that MIG suppresses GDF-15-induced inflammation.

**Discussion:**

This study suggests GDF-15 as a novel PALF outcome biomarker, posits GDF-15 alongside HMGB1 as a central node within the intricate web of systemic inflammation dynamics in PALF, and infers a novel, negative regulatory role for MIG.

## Introduction

Pediatric Acute Liver Failure (PALF) is a complex, rapidly evolving clinical syndrome with diverse etiology that occurs in previously healthy children of all ages (Psacharopoulos et al., 1980; Mondragon et al., 1992). The advent and advancement of pediatric liver transplantation (LTx) has provided a potential life-saving therapeutic option for children with liver derangements. The most common identified etiology for PALF is acetaminophen toxicity mostly due to overdose (APAPo) and while survival is common in this scenario, LTx may be needed and is lifesaving in severely ill patients (Mahadevan et al., 2006). Notably, 45% of PALF cases lack an identified diagnosis, compared to only a 15% incidence of indeterminate ALF in adults (Lee, 2003); children with indeterminate PALF also have lower rates of spontaneous survival and higher rates of death and LTx (Squires et al., 2006). Thus, while prognostic capabilities in PALF are critical, too often the lack of mechanistic knowledge regarding the pathobiology of PALF means that specific prognostic and therapeutic targets to predict death/spontaneous survival are not available.

We have helped address these knowledge gaps by 1) demonstrating three distinct dynamic network archetypes of systemic inflammation associated with spontaneous survival, death, and successful LTx, respectively, showing that the network phenotype of spontaneous survivors was distinct from that of non-survivors but surprisingly similar to that of patients that would go on to receive LTx (Azhar et al., 2013; Zamora et al., 2017); 2) implicating the damage-associated molecular pattern (DAMP) molecule High Mobility Group Box 1 (HMGB1) as a central driver of dynamic pro-inflammatory networks in PALF induced by APAP (Zamora et al., 2019); and, most recently, 3) by defining age-specific inflammatory networks in PALF (Vodovotz et al., 2020).

These studies suggest that defining dynamic networks of inflammation using biological samples from PALF patients along with *in vitro* experimental systems of relevance to PALF can lead to the identification of novel outcome biomarkers and therapeutic targets (Zamora et al., 2019), and a central aspect of this process is the discovery novel inflammatory mediators and their contextual placement in dynamic networks of PALF-associated inflammation. Growth/differentiation factor 15 (GDF-15) is a pleiotropic protein that plays key roles in prenatal development (Lawton et al., 1997), in multiple inflammatory processes (Desmedt et al., 2019), in the regulation of cellular responses to stress signals (Wang et al., 2012), and in tissue repair after acute injury in adult life (Desmedt et al., 2019). Elevated circulating GDF-15 is predictive of mortality in cancer, cardiovascular disease, chronic renal failure, and heart failure (Wiklund et al., 2010). More recently, elevated levels of GDF-15 were reported in children diagnosed with mitochondrial hepatopathy (MH), including some with the PALF phenotype, compared to other childhood liver diseases (Van Hove et al., 2024). The same study concluded that elevated GDF-15, combined with FGF21, might serve as predictor for MH and have prognostic implications. We therefore hypothesized that delineating the dynamic patterns of expression and release of GDF-15 and placing this mediator in a broader inflammatory context could help stratify PALF outcome subgroups.

## Methods

### Selection of PALF patients

The present study was focused on inferring a potential role for GDF-15 in the broader context of PALF-related systemic inflammation associated with spontaneous survival or non-survival, and therefore LTx was not considered explicitly. PALF samples were available through the Pediatric Acute Liver Failure Study Group (PALF; NIH/NIDDKD: 5U01 KD58369). Sample collection was conducted in accordance with both the Declarations of Helsinki and Istanbul, and in accordance with the relevant guidelines and regulations approved by the Institutional Review Boards from all participating institutions listed in our previous publications (Zamora et al., 2017; Zamora et al., 2019; Vodovotz et al., 2020), with written informed consent from parents and/or legal guardians and Certificate of Confidentiality provided by NIH. Entry criteria for the study included children less than 18 years of age with 1) no known evidence of chronic liver disease, 2) biochemical evidence of acute liver injury, and 3) hepatic-based coagulopathy (not corrected with parenteral vitamin K) defined as a prothrombin time (PT) ≥15 s or international normalized ratio (INR) ≥1.5 in the presence of clinical hepatic encephalopathy (HE), or a PT ≥20 s or INR ≥2.0 regardless of the presence or absence of HE. After enrollment, demographic and clinical data were recorded daily for up to 7 days with a single daily serum sample for research scheduled to be collected on the calendar day of enrollment (d0) or with the first morning blood draw following enrollment and daily for up to 7 days (d1-d7), or until death, or discharge from hospital. Serum samples were promptly frozen at −80°C at the enrollment site and later batch-shipped to the research biorepository for long-term storage. Participants were selected if they had at least 3 daily samples with at least 100 µL of serum available. Clinical outcomes were assigned at 21 days following enrollment and patients were segregated into two subgroups: spontaneous survivors without LTx (survivors, S) and non-survivors (NS). Serum samples from participants meeting all criteria were shipped from the NIH biorepository to our Research Laboratory. The detailed clinical criteria and demographics of PALF participants have been reported in our prior studies (Zamora et al., 2017; Vodovotz et al., 2020).

### Vertebrate animals, mouse hepatocyte isolation, and cell culture

Studies in mice were conducted in accordance with an approved animal protocol by the Institutional Animal Care and Use Committee (IACUC) at the University of Pittsburgh. Since working with very young mice (pups) is technically challenging, we utilized hepatocytes (HC) from 8–12 weeks Old mice, an age roughly equivalent to that of adolescents/young adult humans. Primary mouse hepatocytes (HCs) were harvested and processed following previously published methods (Ziraldo et al., 2013; Zamora et al., 2018). The same IACUC-approved protocol (Protocol No. 1105736) was used for both wild-type C57BL/6 mice (procured from Jackson Labs, Bar Harbor, ME) and hepatocyte-specific HMGB1-null mice (HC-HMGB1^−/−^) generated on a C57BL/6 genetic background (Huang et al., 2014). In brief, we exposed the cells to a toxic dose of APAP (10 mM) for varying durations (1, 3, 6, 24, and 48 h). Control cells were incubated in medium alone, as previously described (Zamora et al., 2019). We subsequently assayed the cell supernatants for 23 inflammatory mediators, as detailed in Supplementary Figure S1 and described below.

### Analysis of inflammatory mediators

A total of 28 mediators (26 using a multiplex Luminex™ kit plus HMGB1 and NO_2_^−^ + NO_3_^−^) were assayed in human samples and 23 mediators (20 using a multiplex Luminex™ kit plus HMGB1, NO_2_^−^ + NO_3_^−^ and GDF-15) were assayed in mouse samples, respectively. Multiplexed beadsets were assessed using a Luminex™ 100 IS apparatus (Luminex, Austin, TX) and the Human 25-plex^®^ Luminex™ and 20-plex Milliplex™ Mouse Cytokine/Chemokine Panel I beadsets (Millipore, Billerica, MA). These antibody bead kits include:

Human (26 mediators): Eotaxin, GDF-15, Granulocyte-Macrophage Colony-Stimulating Factor (GM-CSF), Interferon (IFN)-α2, IFN-γ, Interleukin (IL)-1β, IL-1 Receptor Antagonist (IL-1RA), IL-2, soluble IL-2 receptor α chain (sIL-2Rα), IL-4, IL-5, IL-6, IL-7, IL-8, IL-10, IL-12p40, IL-12p70, IL-13, IL-15, IL-17A, IFN-γ-inducible Protein of 10 kDa (IP-10/CXCL10), Monocyte Chemotactic Protein-1 (MCP-1/CCL2), Monokine Induced by γ-Interferon (MIG/CXCL9), Macrophage Inflammatory Protein (MIP)-1α, MIP-1β, and Tumor Necrosis Factor (TNF)-α.

Mouse (20 mediators): GM-CSF, IFN-γ, IL-1α, IL-1β, IL-2, IL-4, IL-5, IL-6, IL-10, IL-12p40, IL-12p70, IL-13, IL-17, IP-10/CXCL10, Keratinocyte-derived Cytokine (KC/CXCL1), MCP-1/CCL2, MIG/CXCL9, MIP-1α/CCL3, TNF-α, and Vascular Endothelial Growth Factor (VEGF).

Human and mouse HMGB1 were assayed using a commercially available ELISA (Shino-Test, Kanagawa, Japan). NO_2_^−^ + NO_3_^−^ were assayed using the nitrate reductase method (Cayman Chemical, Ann Arbor, MI). GDF-15 was measured using a commercially available mouse-specific ELISA kit (LifeSpan BioSciences, Inc., Seattle, WA).

### Statistical and data-driven computational analyses

Two-Way Analysis of Variance (ANOVA) followed by the Holm-Sidak *post hoc* test and Mann-Whitney Rank Sum test were used to analyze the response and time-dependent changes in inflammatory mediators across PALF subgroups and in the mouse HC experiments using SigmaPlot™ 14 (Systat Software, Inc., San Jose, CA) as indicated.

Spearman Correlation Analysis of inflammatory mediators as a function of age and outcome was performed using SigmaPlot™ 14 (Systat Software, Inc., San Jose, CA).

AUC ROC (Area Under The Curve -Receiver Operating Characteristics) curve was calculated using MetaboAnalyst, a web-based tool suite developed for comprehensive metabolomic data analysis (https://www.metaboanalyst.ca).

Volcano plot analysis was performed with a fold change threshold set at 2.0 and significance set at P < 0.05 using MetaboAnalyst.

Dynamic Network Analysis (DyNA) was carried out to define the central inflammatory network nodes as a function of both time and experimental condition or patient subgroup. Using inflammatory mediator measurements of at least three time-points per experimental group, networks were created over consecutive time intervals using MATLAB™ as described previously (Zamora et al., 2017; Zamora et al., 2019; Vodovotz et al., 2020; Ziraldo et al., 2013; Mi et al., 2011; Namas et al., 2016a; Abboud et al., 2016; Sadowsky et al., 2016; Vodovotz et al., 2017; Schimunek et al., 2018; Tohme et al., 2019; Lamparello et al., 2019; Almahmoud et al., 2019; Schimunek et al., 2020). Network connections ([edges], or number of trajectories of mediators that move in parallel [black edges = positive correlations] or in an anti-parallel [red edges = negative correlations] fashion) are created if the Pearson correlation coefficient between any two nodes (inflammatory mediators) at the same time interval is greater or equal to a threshold of an absolute value of 0.85 (to be determined empirically), as indicated. The network complexity for each time interval was calculated using the following formula: Sum (N_1_ + N_2_ +…+ N_n_)/(n−1), where N represents the number of edges/connections for each node/mediator, and n is the total number of mediators analyzed. The total number of connections represents the sum of the number of edges across all time-intervals for all animals or patients in each subgroup.

Dynamic Bayesian Network (DyBN) Inference was carried out using an algorithm adapted from Grzegorczyk & Husmeier (Grzegorczyk and Husmeier, 2011) and implemented in MATLAB^®^ (Azhar et al., 2013; Zamora et al., 2017; Zamora et al., 2019). Given time-series data, DyBN inference provides a means of inferring causal relationships among variables (e.g., inflammatory mediators) based on probabilistic measure. The algorithm uses an inhomogeneous dynamic changepoint model, with a Bayesian Gaussian with score equivalence (BGe) scoring criterion. Notably, DyBNs consider the joint distribution of the entire dataset when making inferences about the dependencies among variables or nodes in the network. The output of the aforementioned algorithm is a final graph structure indicating the interactions. Central/high-feedback nodes are those that exhibit self-feedback in addition to being connected to other nodes.

## Results

### Clinical outcomes in PALF patients

A comprehensive table with detailed demographic and clinical data in three PALF subgroups (survivors, non-survivors and LTx) can be found in a previous publication from our group (Zamora et al., 2017). Key demographics for the study groups utilized here (PALF survivors vs. non-survivors) are shown in Table 1.

**TABLE 1:** Demographic and clinical data for PALF study patients.

PALF study	Survivors (n = 14)	Non-survivors (n = 7)	^a^P
Age (yr.), median [Q1-Q3]	9.74 [1.52–14.75]	0.81 [0.08–7.57]	0.217
Sex, Male, % (n)	50.0% (7)	71.4% (5)	1.000
ALT at enrollment (IU/L), median [Q1-Q3]	2,935 [958–4,869]	104.0 [48–1,671]	0.057
INR at enrollment, median [Q1-Q3]	2.54 [1.91–3.70]	2.40 [2.15–4.60]	1.000
Total Bilirubin at enrollment (mg/dL), median [Q1-Q3]	2.80 [2.30–8.95]	10.60 [4.90–24.3]	0.061
Creatinine at enrollment (mg/dL), median [Q1-Q3]	0.50 [0.40–0.65]	0.40 [0.20–1.25]	0.65
Venous ammonia at enrollment (µmol/L), median [Q1-Q3]	70 [62–83]	164 [102–220]	0.303
Final Diagnosis (n)			
Survivors			
acetaminophen - Acute Toxicity (1)			
acetaminophen - Chronic Exposure (1)			
acetaminophen (not specified) (1)			
Autoimmune hepatitis (2)			
Ischemia/Shock (1)			
Mitochondrial hepatopathy (1)			
Other diagnosis (2)			
Indeterminate (5)			
Non-survivors			
Enterovirus/Coxsackie/Echovirus (1)			
Ischemia/Shock (1)			
Mitochondrial hepatopathy (1)			
Neonatal Hemochromatosis (NH)/GALD (1)			
Veno-occlusive disease (1)			
Indeterminate (2)			

^a^ Comparison of Survivors vs. Non-survivors was performed using *t*-test or Fisher Exact Test (sex) using SigmaPlot™ 14 (Systat Software, Inc., san jose, CA).

### Circulating GDF-15 is elevated significantly in PALF non-survivors vs. spontaneous survivors

**FIGURE 1:**
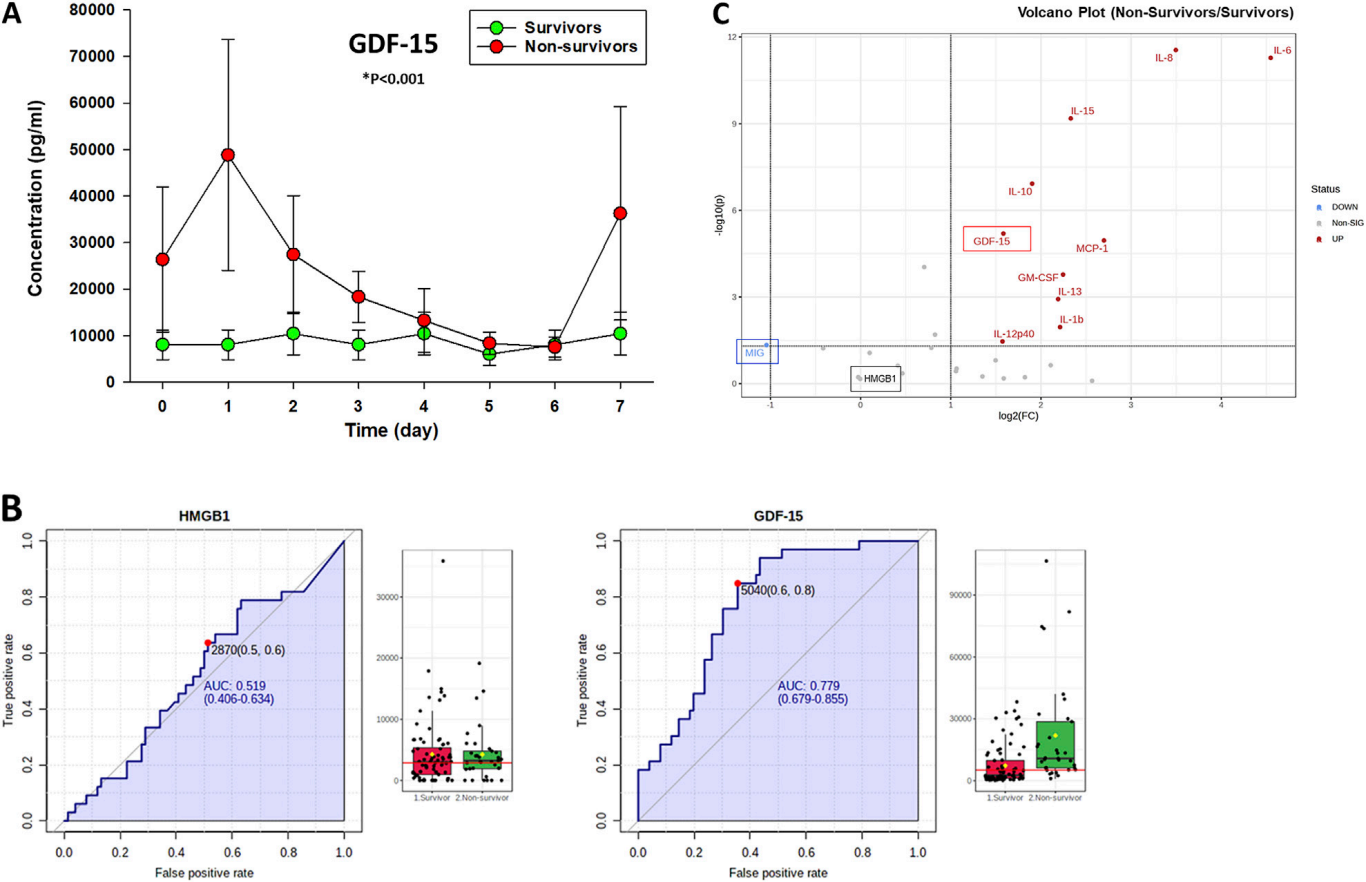
Time-dependent release of GDF-15 in PALF patients. **(A)** Serum samples from PALF patients [spontaneous survivors (n = 14) and non-survivors (n = 7)] were assessed for GDF-15 using Luminex technology as described in Materials and Methods*.* Results represent the mean ± SEM, analyzed by Mann-Whitney Rank Sum test (*P < 0.001) as described. **(B)** Analysis of AUC ROC for HMGB1 and GDF-15 in PALF suggests that GDF-15 levels might be more helpful in prognosticating survival. Fig. shows the ROC curves and serum levels (survivors vs. non-survivors) of HMGB1 (left) and GDF-15 (right) calculated using MetaboAnalyst as described in Materials and Methods. The black dots represent the concentrations of the selected feature (e.g., HMGB1 protein) from all samples (all time points) in each patient group. The notch indicates the 95% confidence interval around the median of each group, defined as ± 1.58*IQR/sqrt(n). The mean concentration of each group is indicated with a yellow diamond. The horizontal red line represents an optimal cutoff calculated automatically by the algorithm. **(C)** Volcano Plot Analysis of statistical significance vs. magnitude of change in the inflammatory mediator data obtained by Luminex™ shows GDF-15 as one of the top genes that surpassed the fold change threshold (set at 2.0) and exhibited the smallest P-values (significance set at *P* < 0.05).

We previously assessed a broad panel of mediators that represent most of the major inflammatory and immune pathways in serum samples of PALF patients (Zamora et al., 2017). For this study and based on sample availability, serum samples from a selected number of PALF individuals (spontaneous survivors (n = 14) and non-survivors (n = 7) were assessed for several inflammatory mediators including GDF-15 as described in *Materials and Methods*. The comparison of those mediator time-courses in survivors vs. non-survivors (significance set at *P* < 0.05) is shown in Supplementary Figure S2. As shown in Figure 1A, GDF-15 levels were also elevated significantly in PALF non-survivors compared to survivors. Moreover, in both subgroups, these levels were significantly higher than the circulating levels reported in healthy children (Montero et al., 2016), (Galuppo et al., 2022) (200–400 pg/mL) and children with other known liver diseases (Van Hove et al., 2024). Furthermore, analyses of AUC ROC for HMGB1 and GDF-15 (non-survivors vs. survivors) in PALF suggested that while high levels of HMGB1 and GDF-15 are found in both patient subgroups, GDF-15 levels might be more helpful in prognosticating survival [AUC: GDF-15 (0.779) vs. HMGB1 (0.519)] (Figure 1B). A Volcano plot analysis comparing non-survivors to survivors highlighted that, of the mediators upregulated in non-survivors, GDF-15 was among the top mediators that both exceeded the fold change threshold of 2.0 and exhibited the smallest P-values (*P* < 0.05) when comparing non-survivors and spontaneous survivors (Figure 1C). Interestingly, this analysis also revealed that MIG/CXCL9, which is downregulated in non-survivors compared to survivors, serves as the sole assessed mediator capable of distinguishing between these two groups. In line with the AUC-ROC results (Figure 1B), HMGB1 did not differentiate between NS and S (Figure 1C).

Based on our previous work (Vodovotz et al., 2020), it would be expected that age can be associated significantly with inflammatory markers as well as clinical outcomes. To determine if levels of HMGB1, GDF-15 and MIG are correlated with age, we performed a correlation analysis (all samples, all time points) of age vs. those mediators (Table 2). Interestingly, we found that age was significantly anti-correlated with GDF-15 but not with HMGB1 or MIG (Table 2, top). Furthermore, performing the correlation analysis as a function of both age and outcome revealed that the anti-correlation of GDF-15 vs. age was mostly due to the significant negative correlation observed in S as compared to NS. This latter analysis also revealed that HMGB1 was significantly anti-correlated with age only in NS but not in S (Table 2, bottom). We note that these correlation results should be interpreted cautiously due to the small number of patients as discussed below.

**TABLE 2:** Spearman Rank Correlations Age vs. mediator.

Mediator		Correlation coefficient		^a^P-value		No. Of samples	
HMGB1		−0.0705		0.466		109	
GDF-15		−0.448		<0.0001		109	
MIG		0.0518		0.592		109	

Age vs. mediator as a function of outcome							
Mediator	Outcome		^a^P-value		^a^P-value		No. Of samples
HMGB1	survivor		0.0407		0.727		76
	non-survivor		−0.347		0.0479		33
GDF-15	survivor		−0.39		0.000537		76
	non-survivor		−0.113		0.53		33
MIG	survivor		−0.2		0.0836		76
	non-survivor		0.108		0.548		33

^a^ Analysis was performed using SigmaPlot™ 14 (Systat Software, Inc., san jose, CA).

### Dynamic bayesian network (DyBN) inference identifies both common and distinct nodes of PALF-associated systemic inflammation

**FIGURE 2:**
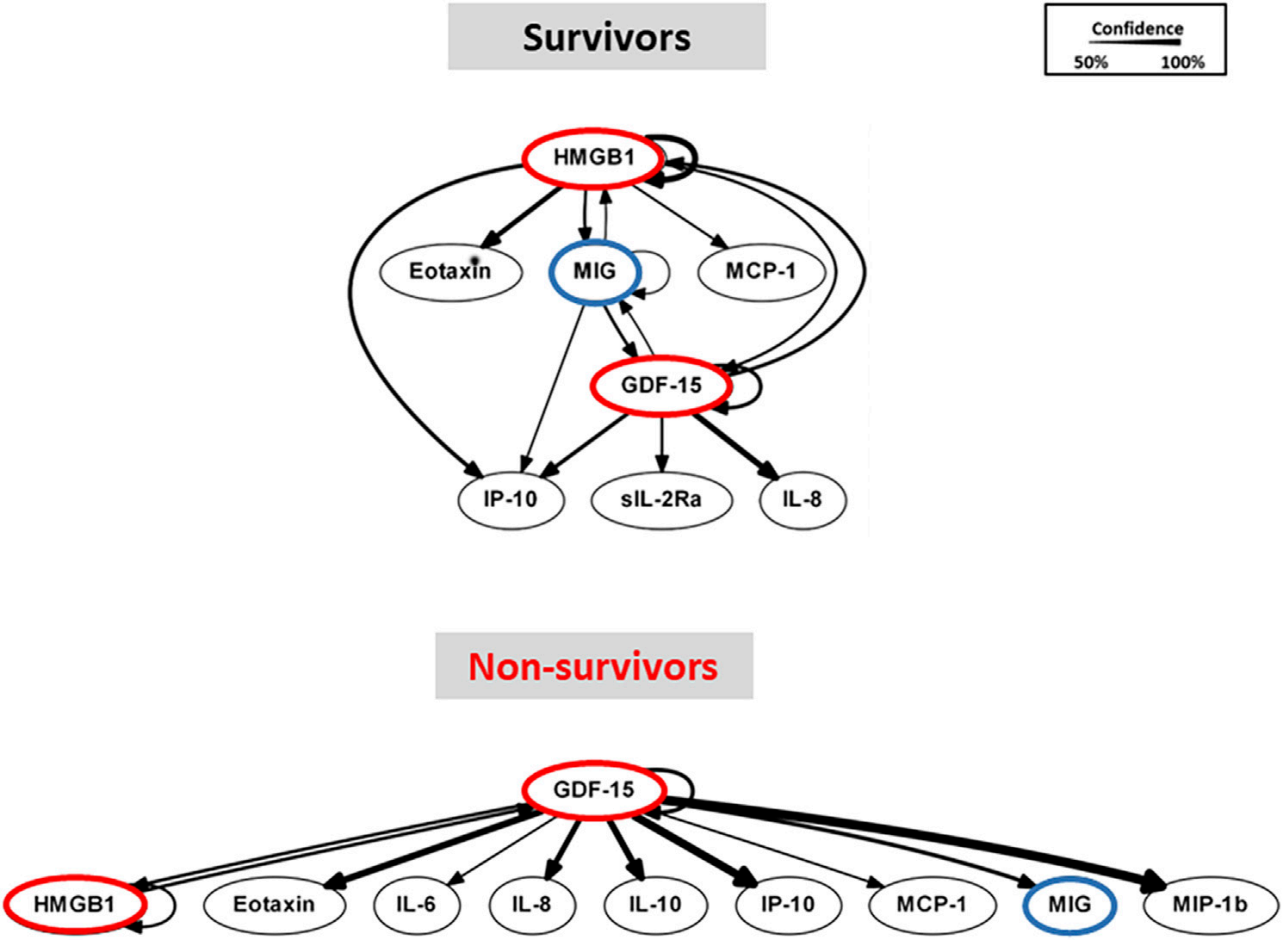
Dynamic Bayesian Network (DyBN) analysis of circulating inflammatory mediators in PALF patients. Circulating inflammatory mediators in serum samples from PALF spontaneous survivors (S, n = 14 patients) and non-survivors (NS, n = 7 patients) were measured and DyBN analysis was performed as described in *Materials and Methods*. Inflammatory mediators are shown as nodes, and the arrows connecting them suggest an influence of one mediator on the one(s) to which it is connected. The arrows do not distinguish positive from negative influences of one mediator on another. Semi-circular arrows suggest either positive or negative feedback of a given mediator on itself.

As in our prior studies, we utilized DyBN inference to determine if mediator feedback structures in inflammatory networks in PALF are related to clinical outcomes. Similar to our previous studies in PALF, trauma, and sepsis (Azhar et al., 2013; Zamora et al., 2017; Ziraldo et al., 2013; Almahmoud et al., 2015; Namas et al., 2016b), we focused on mediators that exhibit self-feedback as central nodes in both PALF subgroups (survivors vs. non-survivors), hypothesizing that such self-feedback nodes represent possible regulatory mechanisms for self-sustaining inflammation (Namas et al., 2015; Voit et al., 2023). Notably, though data were segregated by outcome before being subjected to DyBN inference, the algorithm made no assumptions regarding the connectivity of the network in any subgroup. This analysis suggested a primary network driven by two core motifs: HMGB1 and GDF-15, with both mediators inferred to exhibit self-feedback motifs (Figure 2), thus positioning GDF-15 alongside HMGB1 (Zamora et al., 2017; Zamora et al., 2019; Vodovotz et al., 2020) as a central node in dynamic networks of PALF-associated systemic inflammation. Notably, a key difference between the DyBN networks of spontaneous survivors and non-survivors is MIG, which links HMGB1 and GDF-15 in the former but not in the latter (Figure 2). This finding led us to hypothesize a potential role for MIG in dampening the effects of HMGB1 in PALF non-survivors, and we explored this hypothesis using additional computational analyses.

### Dynamic connectivity of GDF-15 in protein-level inflammation networks associated with key clinical PALF outcomes

**FIGURE 3:**
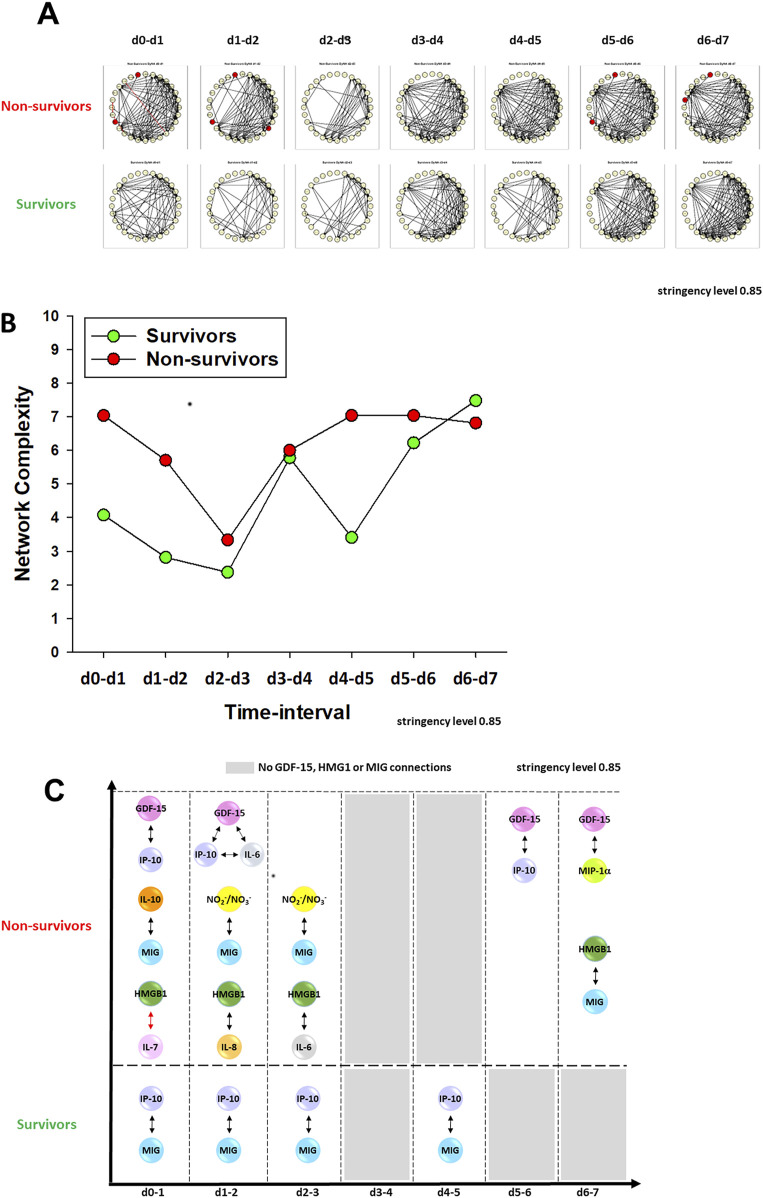
Dynamic Network Analysis (DyNA) of inflammatory mediators in PALF patients. **(A)** An overview of all the dynamic networks and mediator connections over seven time-intervals (d0-d1, d1-d2, d2-d3, d3-d4, d4-d5, d5-d6, d6-d7) of two PALF patient subgroups determined by DyNA (stringency level 0.85) as described in *Materials and Methods*. Closed red circles represent mediators directly connected to GDF-15. Black and red lines connecting two mediators represent positive and negative correlations, respectively. Panel **(B)** shows the complexity of networks shown in Panel **(A)** and Panel **(C)** highlights the detailed HMGB1, GDF-15 and MIG connectivity resulting from DyNA in PALF non-survivors and survivors calculated as described in *Materials and Methods*.

We have demonstrated previously that PALF non-survivors have more robust dynamic networks of inflammation than those of survivors, in line with the concept of pathology driven by self-sustaining inflammation (Zamora et al., 2017; Zamora et al., 2019; Vodovotz et al., 2020). Accordingly, we next hypothesized that the connectivity of GDF-15, as previously shown for HMGB1 (8), will be characteristic of each patient subgroup outcome and might serve to differentiate among them. We utilized Dynamic Network Analysis (DyNA) (Mi et al., 2011; Namas et al., 2015; Voit et al., 2023) to delineate the temporal progression of inflammation networks in a granular manner. As demonstrated in our previous studies (Zamora et al., 2017; Zamora et al., 2019), PALF non-survivors exhibited more complex inflammation networks compared to those observed in spontaneous survivors (Figures 3A, B), in line with the hypothesis that self-sustaining inflammation drives PALF pathobiology. DyNA also suggested differential network connectivity in non-survivors compared to survivors. In non-survivors, GDF-15 was connected to IL-6 (d0-d2) and the chemokines IP-10/CXCL10 (d0-d1, d5-d6) and MIP-1α/CCL3 (d6-d7); however, GDF15 exhibited no inflammatory network connections in survivors. Similarly, in non-survivors, HMGB1 was connected to IL-7 (d0-d1), IL-8 (d1-d2), IL-6 (d2-d3), and MIG (d6-d7); like GDF15, HMGB1 was not connected to other assessed mediators in survivors (Figure 3C). This analysis also revealed similarities between the profiles of GDF-15 and HMGB1: both exhibited elevated expression but low connectivity in non-survivors. We have hypothesized previously that mediators present at high systemic levels but with low interconnectivity might represent pathological inflammatory processes and could serve as potential disease biomarkers (Mi et al., 2011; Zamora et al., 2019). Interestingly, the connectivity of MIG was also different in PALF non-survivors vs. survivors: in the former, MIG was connected to IL-10 (d0-d1), NO_2_^−^/NO_3_^−^ (d1-d3), and HMGB1 (d6-d7), whereas in the latter it was connected solely to IP-10 (Figure 3C).

### Circulating levels of GDF-15 in PALF patients and primary mouse hepatocytes in the context of APAP toxicity

**FIGURE 4:**
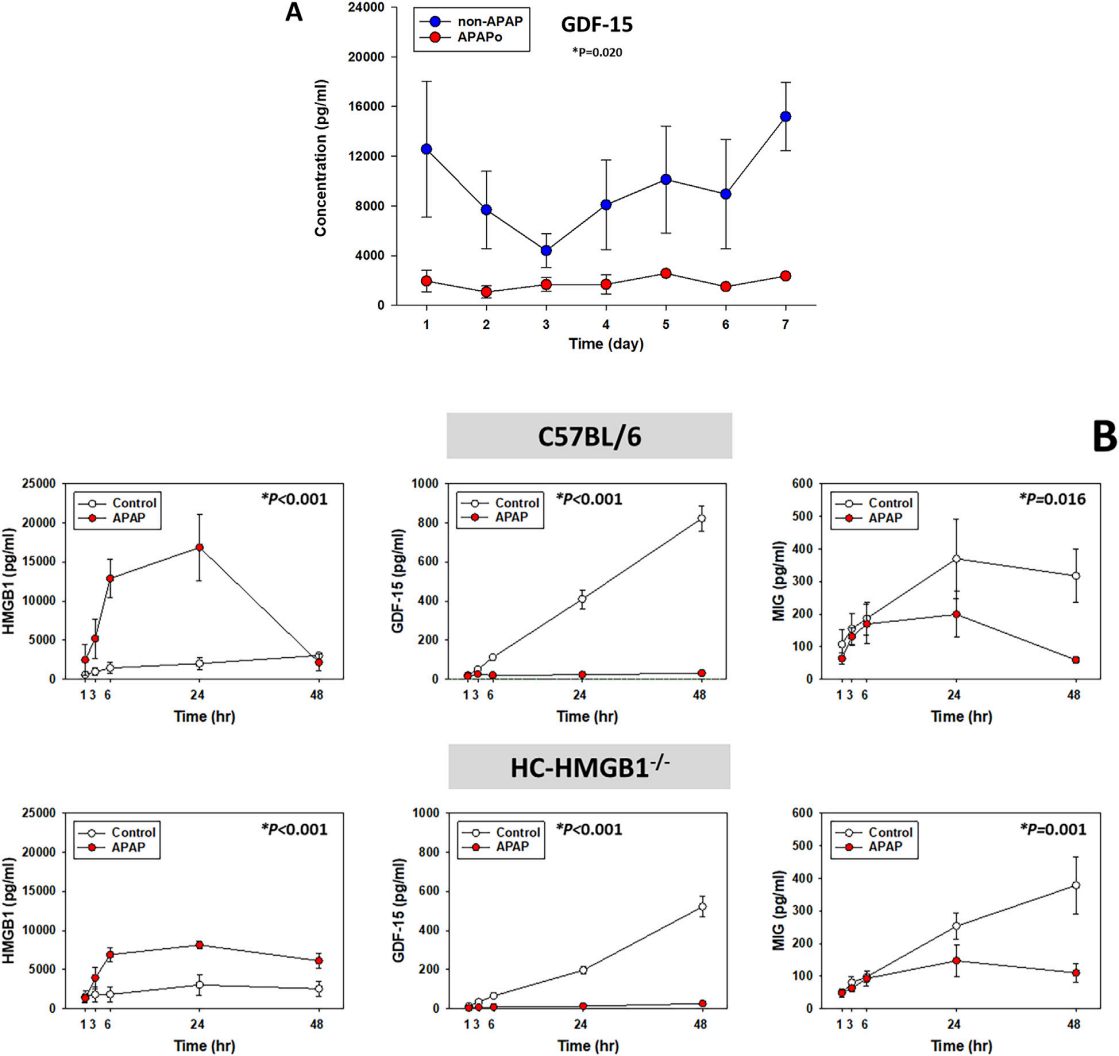
Circulating levels of GDF-15 in PALF patients and primary mouse hepatocytes in the context of APAP toxicity. **(A)** Time‐dependent release of GDF-15 in PALF survivors diagnosed with APAPo (n = 3) or non-APAP (n = 11) as described in Figure 1 and analyzed by Mann-Whitney Rank Sum test (*P < 0.05) as described. **(B)** Supernatant levels of HMGB1, GDF-15 and MIG (measured using Luminex technology as described in Materials and Methods) in cultures of mouse hepatocytes treated with 10 mM APAP for the times indicated. Cells were from n independent experiments/animas as follows: C57BL/6 [Control (n = 3), APAP (n = 3); HMGB1^−/−^: Control [n = 4], APAP (n = 5)]. Results represent the mean ± SEM, analyzed by Two‐Way ANOVA (*P < 0.05).

We have identified HMGB1 previously as a central driver of dynamic pro-inflammatory networks in both acetaminophen (APAP)-induced PALF and in APAP-treated mouse hepatocytes (HCs) *in vitro* (Zamora et al., 2019). Hypothesizing that GDF15 might also play a role in such networks, we assessed the levels of GDF-15 in the systemic circulation of PALF patients diagnosed with either APAP overdose (APAPo) or non-APAP (all survivors). Despite the relatively low number of patients in both subgroups, we observed a statistically significant difference in GDF-15 levels between APAPo and non-APAP patients (Figure 4A). This finding suggests that APAPo may decrease or interfere with the concentration of GDF-15 in the circulation of PALF patients. While no significant differences in major injury markers were observed between the two patient subgroups (Supplementary Figure S3), it is important to note that non-APAP patients were notably younger than APAPo individuals. Consequently, as observed in other studies, including ours (Zamora et al., 2019) (Van Hove et al., 2024), we cannot rule out the possibility that age may play a determining role in the observed effect.

We next thought to investigate the relationship between HMGB1 and GDF-15 in the context of APAP toxicity. For this purpose, we utilized primary HC isolated from wild-type (C57BL/6) and HC-specific HMGB1-null mice (HC-HMGB1^−/−^) following established protocols (Zamora et al., 2019; Ziraldo et al., 2013; Abboud et al., 2016; Tohme et al., 2019). Analyzing the time-courses of 23 inflammatory mediators using Two-Way ANOVA, we observed distinct trajectories depending on both the presence of HMGB1 and APAP treatment (Supplementary Figure S1).

As shown previously (Zamora et al., 2019), we observed a significant elevation in released HMGB1 in APAP-treated wild-type HC compared to control cells, whereas HC-HMGB1^−/−^ cells released a lower level than wild-type HC, although the difference was not statistically significant. Notably, some form of HMGB1 or anti-HMGB1-reactive molecule was still detected in the supernatants of these cell cultures (Figure 4B), in line with previous studies (Yang et al., 2005; Yang et al., 2012; Zamora et al., 2019). Notably, under APAP conditions, the absence (or reduced levels) of HMGB1 was associated with reduced secretion of GDF-15 protein in both cell types (Figure 4B), suggesting a potential cross-regulation between these two mediators. Also, after APAP treatment HC-HMGB1^−/−^ cells had significantly lower levels of GDF-15 (median: 8.75 pg/mL) as compared to wild-type hepatocytes (median: 22.19 pg/mL) (*P = 0.032, analyzed by Kruskal–Wallis ANOVA on Ranks). Interestingly, APAP also affected the secretion of MIG in both wild type and HC-HMGB1^−/−^ HC, suggesting that the decreasing effect of APAP on MIG is independent of HMGB1 (Figure 4B).

### Impact of APAP and endogenous HMGB1 on dynamic inflammatory connectivity of GDF-15 to other inflammatory mediators secreted by mouse hepatocytes *in vitro*

**FIGURE 5:**
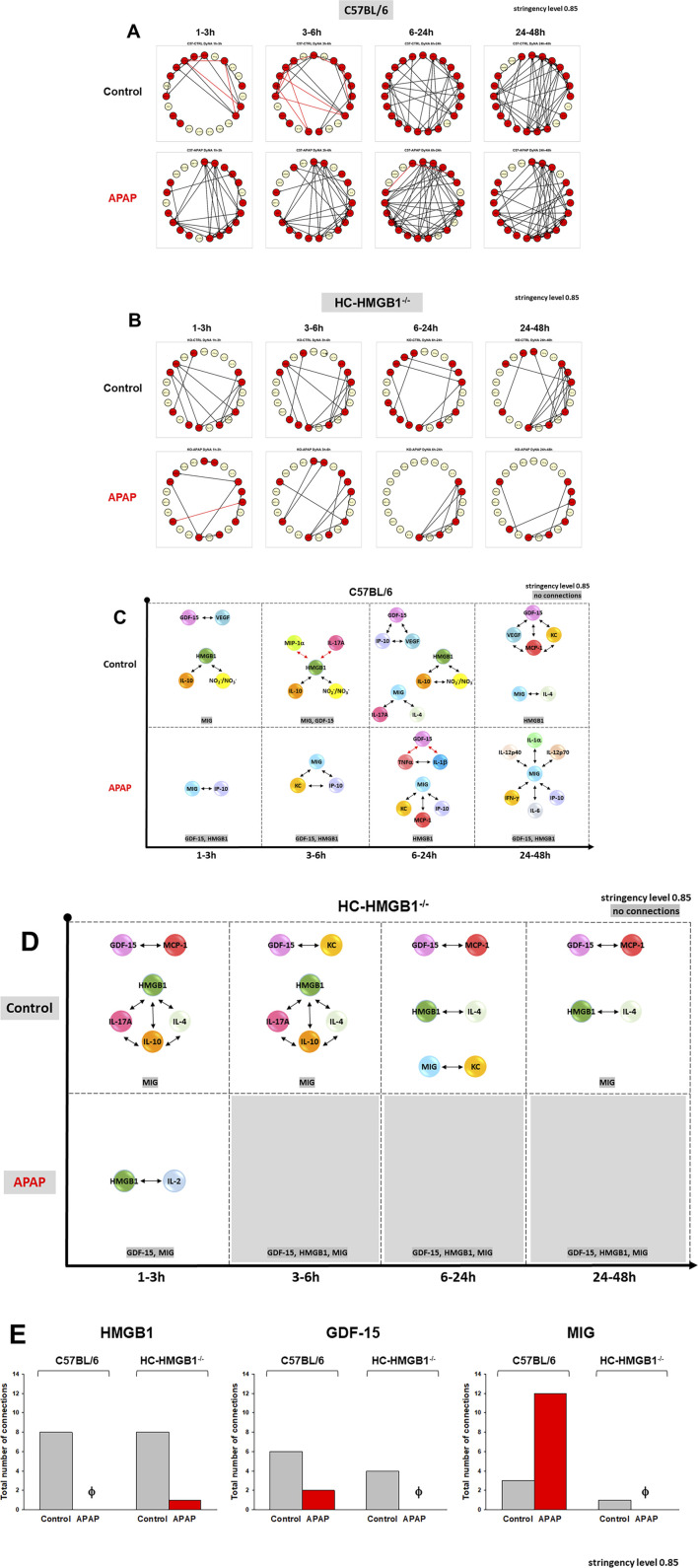
Dynamic Network Analysis (DyNA) of inflammatory mediators in mouse hepatocytes. Cell supernatants from freshly isolated mouse hepatocytes from C57BL/6 mice or HC-HMGB1^−/−^ with or without 10 mM APAP treatment for 1–48 h were assayed for 23 inflammatory mediators as described in *Materials and Methods*. Panels **(A, B)** show an overview of all the dynamic networks (stringency level 0.85) and mediator connections over four time-intervals (1–3, 3–6, 6–24, and 24–48 h) for control and APAP-treated C57BL/6 HCs and HC-HMGB1^−/−^, respectively. Red and yellow circles represent mediators connected to other mediators and mediators without specific connections, respectively. Panels **(C, D)** highlight the detailed GDF-15 connectivity resulting from DyNA in C57BL/6 HCs and HC-HMGB1−/−, respectively. Panel **(E)** displays the total number of connections for HMGB1, GDF-15 and MIG across all experimental groups. These connections were determined and calculated according to the methods outlined in the *Materials and Methods* section.

We next compared the dynamic network patterns of inflammatory mediators in isolated HC from C57BL/6 and HC-HMGB1^−/−^ mice using DyNA. The quantification of DyNA network complexity revealed a significant difference in the response of HC to APAP between the two mouse strains: wild type HC exhibited a much more complex network pattern and a higher total number of network connections as compared to HC-HMGB1^−/−^ cells (117 vs. 53, respectively) (Figures 5A, B; Supplementary Figure S4). Interestingly, in the presence of APAP, DyNA suggested an increased number of total connections in C57BL/6 cells (153) as opposed to a reduced number in HC-HMGB1^−/−^ cells (Galuppo et al., 2022) when compared to cells without treatment (Supplementary Figure S4).

We subsequently directed our attention to the specific connectivity of GDF-15 and visualized the individual connections over time and under different treatments (Figures 5C–E). In wild-type C57BL/6 HCs, GDF-15 exhibited connections with VEGF, IP-10, MCP-1, and KC (Figure 5C). However, in HC-HMGB1^−/−^cells, GDF-15 was connected to MCP-1 and KC only (Figure 5D). Specifically, DyNA network connectivity of GDF15 was reduced in APAP-treated cells as follows: in C57BL/6 cells (Figure 5C), GDF-15 was connected to TNFα and IL-1β (both negative connections), and in HC-HMGB1^−/−^ cells (Figure 5D), no connections were observed. Analysis of the total connectivity of all assessed inflammatory mediators linked directly to GDF-15 revealed the following: in C57BL/6 HC, APAP treatment was associated with decreased connectivity of HMGB1 and GDF-15 in contrast with increased connectivity to MIG (Figure 5E). Conversely, in HC-HMGB1^−/−^ cells, APAP decreased the connectivity of these mediators.

In summary, our analysis revealed that HMGB1 not only affects the expression and release of GDF-15 but also modulates its connectivity to other mediators, an effect that is influenced by exposure to APAP.

## Discussion

Predicting PALF outcomes is complicated, in part because etiology is distinctly different from that of adults (Narkewicz et al., 2018). Several prognostic models have been published using more common biomarkers (Liu et al., 2006; Lal et al., 2020; Lee et al., 2020; Li et al., 2024; Ascher-Bartlett et al., 2024); however, these studies have not led to definitive identification of therapeutic targets. Furthermore, the inclusion of variables that can be subjective and difficult to assess (e.g., encephalopathy) or modifiable by supportive measures such as blood product administration (e.g., PT/INR, fibrinogen), extracorporeal support devices (e.g., ammonia, bilirubin, lactate), or replacement supplementation (e.g., phosphorus, albumin) continues to limit their clinical impact.

PALF-associated immune dysregulation is a principal mechanism for driving organ failure regardless of diagnosis (Zamora et al., 2017; Bucuvalas et al., 2013; Chapin et al., 2018). Studies across diverse diseases point to the complex, dynamic, feedback-dependent nature of inflammation and immunity (Medzhitov, 2021; Vodovotz, 2023), and like in other pathologies there is a need to define central drivers of immune dysregulation in a PALF. We therefore sought to apply computational modeling methods to PALF samples and *in vitro* experimental systems of relevance to PALF to identify immune/inflammatory networks that will define novel biological mechanisms and hopefully help improve clinical care for PALF patients. In the present study, this approach led us to define GDF-15 as a potential biomarker of adverse outcomes such as mortality in PALF, implicated GDF-15 and HMGB1 as central mediators of PALF-associated inflammation, and further suggested that MIG/CXCL9 might attenuate the GDF15-dependent inflammation.

The etiology of PALF and its different clinical manifestations are overly complex and still poorly understood. APAP toxicity, commonly due to APAP overdose (APAPo) is the most common identifiable cause of ALF in both children (Leonis et al., 2013) and adults (Khan and Koppe, 2018). Among many other functions, the liver plays a critical role in inflammation and innate immunity in response to stress, processes that are controlled by HCs, Kupffer cells, and other non-parenchymal cells. Hepatocytes constitute the largest pool of parenchymal cells (approximately 60%–80% of the total liver cells) and are widely used to study liver function *in vitro*. Our initial *in vitro* and *in silico* studies led us to identify HMGB1 as a central mediator of inflammation in PALF (8). Liver injury due to APAPo occurs when inherent mechanisms to detoxify APAP–including conjugation with glutathione–are overwhelmed, resulting in the formation of reactive oxygen species that form destructive adducts with vital intracellular proteins. N-acetyl cysteine (NAC) serves to replete glutathione stores and is the established treatment for acute APAP toxicity (Squires et al., 2013). In that respect, we have demonstrated previously that dynamic Inflammatory networks, and particularly HMGB1 connectivity, were associated with the use of NAC in the context of APAPo (Zamora et al., 2019).

In the present study, in addition to HMGB1, we focused on the Growth Differentiation Factor 15 (GDF-15), also known as macrophage inhibitory cytokine 1 (MIC-1), a multifaceted protein that belongs to the transforming growth factor-β (TGF-β) superfamily of proteins. Physiological GDF-15 levels are low to absent in healthy individuals in most tissues that can show inducible GDF-15 expression, based on a proteomic, multi-tissue map of the human tissue proteome (Uhlén et al., 2015). Recent studies have implicated GDF-15 in energy metabolism, bodyweight control, and appetite regulation through GDF15/GFRAL signaling (Guo et al., 2024; Fejzo et al., 2018; Day et al., 2019; Chang et al., 2020). GDF-15 has also been associated with multiple inflammatory processes, with the regulation of cellular responses to stress signals, and with tissue repair after acute injury in adult life. Serum GDF-15 levels are elevated significantly in critically ill patients, associated with sepsis, organ failure, and disease severity; furthermore, high GDF-15 levels at the time of admission to the intensive care unit (ICU) can predict short- and long-term mortality risk (Buendgens et al., 2017). In a large cohort of more than 800 patients, GDF15 was 94% sensitive and 67% specific for the detection of a significant liver fibrosis (Bellan et al., 2018). In older adults, elevated GDF15 levels were associated with signs of accelerated aging and with poorer recovery after acute illness (Tavenier et al., 2021). Those studies have been in adults; the role of GDF-15 in inflammatory processes in children remains to be elucidated and is thus significant.

Previously, we demonstrated that PALF patient subgroups, with varying outcomes (Zamora et al., 2017), or those with the same outcome but different treatments (Zamora et al., 2019), exhibit distinct dynamic networks of inflammation. These networks were inferred using DyNA, an algorithm designed to define granular network connections over discrete time intervals (Zamora et al., 2017; Zamora et al., 2019; Vodovotz et al., 2020; Ziraldo et al., 2013; Mi et al., 2011; Namas et al., 2016a; Abboud et al., 2016; Sadowsky et al., 2016; Vodovotz et al., 2017; Schimunek et al., 2018; Tohme et al., 2019; Lamparello et al., 2019; Almahmoud et al., 2019; Schimunek et al., 2020). DyNA allowed us to analyze and compare the interconnections among inflammatory mediators within different patient subgroups. We also explored whether mediator feedback structures within inflammatory networks in patients with PALF are indicative of patient outcomes. To achieve this, we employed DyBN inference (Azhar et al., 2013; Zamora et al., 2017; Zamora et al., 2019; Grzegorczyk and Husmeier, 2011), integrating data to define a comprehensive graph that captures the dynamics and potential feedback structures within each patient subgroup. The present results build upon these earlier studies, demonstrating that GDF-15 is both elevated and differentially connected to other inflammatory mediators in PALF non-survivors as compared to survivors. Specifically, our findings suggest that the network connectivity between GDF-15 and HMGB1 is associated with, and may drive, these divergent outcomes. Our *in vitro* studies show that, similar to HMGB1, the connectivity of GDF-15 is influenced by APAP toxicity. Moreover, the differential expression of GDF-15 is largely dependent on the presence of HMGB1 in the context of APAP-induced hepatocyte injury. An intriguing hypothesis, which undoubtedly warrants further investigation in future studies, is that GDF-15-expressing immune cells (Harb et al., 2020) may play a pivotal role in the transit and potential bidirectional transmission of APAP-induced inflammation between the bloodstream and the liver over time. While our results point to a significant connection, they do not confirm the mechanism definitively, and thus additional research is needed to fully elucidate the dynamics of this process.

Our studies pointed to a key mediating role for MIG/CXCL9 in PALF-associated networks of systemic inflammation. Based on DyBN inference, this chemokine linked HMGB1 and GDF-15 directly in survivors. However, MIG appeared downstream of GDF-15 but separate from a module involving GDF-15 and HMGB1 in DyBN networks of non-survivors. Taken together, these findings lead us to hypothesize that MIG helps attenuate the inflammatory response in PALF, and that its absence from the pathways involving GDF15-HMGB1 interplay allows for (or drives) progressively increasing inflammation. We had hypothesized a similar role for MIG in the context of a central network of chemokines involving MIG, MCP-1/CCL2, and IP-10/CXCL10 in critically ill trauma patients (Azhar et al., 2021), and further implicated a role for neural regulation of MIG via the vagus nerve in a recent study in mice (Shah et al., 2024). These prior studies have led us to hypothesize that interactions among these various chemokines help determine the ultimate character of a given acute inflammatory response. Interestingly, PALF survivors’ DyNA and DyBN networks exhibit connectivity between MIG and IP-10, and APAP is associated with upregulation of CXCR3, the receptor for both MIG and IP-10 in mouse models of liver injury (Bone-Larson et al., 2001; Song et al., 2019). In addition, mice lacking CXCR3 displayed augmented liver damage associated with increased expression of HMGB1 protein (Zaldivar et al., 2012) while another study demonstrated that HMGB1 promotes CXCR3 production (Gao et al., 2019). Our findings suggest that a program centered around the axis HMGB1-(MIG)-GDF-15 (and its interactions with IP-10, though this deserves further study) might serve as a signature of liver stress and damage.

It bears repeating, as discussed in our previous publication (Zamora et al., 2019), that in the *in vitro* experiments the levels of HMGB1 were significantly reduced in APAP-treated HC-HMGB1^−/−^ cells, yet some form of HMGB1 or HMGB1 reactive molecule was detected in the supernatants of those cultures. This may be due to differential processing of HMGB1, or to a small contamination with non-HC cells in our primary cultures, or to differentially post-translationally modified forms of HMGB1. For example, hyperacetylation of HMGB1 shifts its equilibrium from a predominant nuclear location toward a cytosolic and subsequent extracellular presence (Yang et al., 2005). In addition, the extracellular activity of HMGB1 as inflammatory mediator is closely related to the redox state of its three key cysteine residues (Yang et al., 2013). Under strong oxidizing conditions such as those seen with APAP exposure *in vitro*, the oxidation of some or all of the cysteine residues could lead to loss of biological activity of HMGB1. Currently, we do not have a definitive answer to that question and future studies will need to address the potential role of these HMGB1 modifications in coordinating networks of inflammation. Nonetheless, in our system the overall effect of APAP is likely not due to the presence of a pro-inflammatory form of HMGB1, as evidenced by the distinct and low network complexity in HC-HMGB1^−/−^ cells as compared to wild-type cells.

There are multiple other limitations to our study, key among them the fundamental principle that correlation does not imply causality. Network analyses, while powerful tools for visualizing relationships among variables, inherently lack mechanistic insights. However, these data-driven modeling approaches can serve as bridges for inferring underlying mechanisms (Aerts et al., 2014; Alber et al., 2019), e.g., to MMP14, as we have demonstrated previously (Abboud et al., 2016). In this context, an intriguing aspect of GDF-15 biology pertains to its functional interactions with other proteins that potentially contribute to liver injury and inflammation. Among the proteins implicated in this intricate network, matrix metalloproteinase MMP14/MT1 (Sato et al., 1994; Bassiouni et al., 2021) is one of the few shown to be cleaved by and interact with GDF-15, although the precise implications of this interaction remain elusive. MMP14 has been involved in inflammation and tissue repair (Murphy, 2017). Notably, hepatic MMP14 expression is elevated in pediatric liver transplant recipients (Voutilainen et al., 2017), yet the activation of MMP14 and its specific interplay with GDF-15 in the context of PALF remain unexplored. Moreover, GDF-15-mediated biology does not always hinge on the recently identified receptor, GFRAL (Mullican et al., 2017; Wischhusen et al., 2020; Chow et al., 2022). Another limitation of our studies is that the interpretation of our modeling results is constrained by our imperfect current understanding of the mechanistic underpinnings of PALF. Consequently, we cannot assert that we capture all crucial interactions among inflammatory mediators. This limitation arises from the DyNA or DyBN algorithms’ inability to fully represent key dynamic interactions in both clinical and experimental models of PALF. Studying a larger panel of inflammatory mediators may well shed further insights by changing the network structures associated with PALF subgroups. Additionally, we acknowledge that PALF is considered as a rare disease, resulting in a limited pool of patients available for clinical research.

In conclusion, the significance of this study lies in the computational delineation of novel, intertwined circuits and common dynamic network patterns involving GDF-15, HMGB1, and MIG in the context of inflammation and liver failure. Collectively, these data highlight the complex relationship between inflammatory mediators and liver diseases, positioning GDF-15 and HMGB1 as central mediators of hepatic and systemic inflammation in PALF and further recasting MIG as a chemokine that may help control inflammation.

## Data Availability

The datasets presented in this study can be found in online repositories. The names of the repository/repositories and accession number(s) can be found below: Data are available upon request from RZ and YV at the Surgery Research Laboratory, University of Pittsburgh, as well as from the NIDDK Central Repository (NIDDK-CR) website, Resources for Research (R4R), https://repository.niddk.nih.gov/.

## Abbreviations

APAP, acetaminophen; DyBN, Dynamic Bayesian Network; DyNA, Dynamic Network Analysis; Granulocyte-Macrophage Colony-Stimulating Factor, GM-CSF; Growth/Differentiation Factor-15, GDF-15; HMGB1, Hepatocyte(s), HC(s); High-Mobility Group Box 1; Hepatocyte-specific HMGB1-null mice, HC-HMGB1^−/−^; Interferon, IFN; IL, Interleukin; IP-10/CXCL10, IFN-γ-inducible Protein of 10 kDa; IL-1RA, IL-1 Receptor Antagonist; sIL-2Rα, soluble IL-2 receptor α chain; KC/CXCL1, Keratinocyte-derived Cytokine; MCP-1/CCL2, Monocyte Chemotactic Protein-1; MIG/CXCL9, Monokine Induced by γ-Interferon; MIP, Macrophage Inflammatory Protein; PALF, Pediatric Acute Liver Failure; TNF, Tumor Necrosis Factor; VEGF, Vascular Endothelial Growth Factor.
