# Supplementary material for: Intertwined roles for GDF-15, HMGB1, and MIG/CXCL9 in Pediatric Acute Liver Failure
Source: Front Syst Biol. 2024 Oct 15;4:1470000. doi: 10.3389/fsysb.2024.1470000 (PMC12342023; doi:10.3389/fsysb.2024.1470000)
Supplement: Supplementary file 1 [file DataSheet1.PDF]

Supplementary Figures

Intertwined roles for GDF-15, HMGB1, and MIG/CXCL9 in Pediatric Acute Liver Failure

Ruben Zamora[1,2,3], Jinling Yin[1], Derek Barclay[1], James E. Squires[4], Yoram Vodovotz[1,2,3*]

[1]Department of Surgery, University of Pittsburgh, Pittsburgh, PA 15213, USA; [2]Center for Inflammation and Regenerative Modeling, McGowan Institute for Regenerative Medicine, Pittsburgh, PA 15219, USA; [3]Pittsburgh Liver Research Center, University of Pittsburgh, Pittsburgh, PA 15213, USA; [4]Department of Pediatrics, University of Pittsburgh, Pittsburgh, PA 15213, USA

Supplementary Figure 1 (Fig. S1). Time-courses of 23 inflammatory mediators in samples from mouse hepatocytes. Supernatants from freshly isolated mouse hepatocytes from C57BL/6 mice or HC-HMGB1$^{-/-}$ with or without APAP treatment for 1-48h were assayed for 23 inflammatory mediators as described in *Materials and Methods*. Results represent the mean ± SEM, analyzed by Two-Way ANOVA (*P<0.05).

Supplementary Figure 2 (Fig. S2). Comparison of time-courses of 28 inflammatory mediators in serum samples from PALF patients. Serum samples (d0-d7) from survivors (n=24) and non-survivors (n=7) were assayed for 28 inflammatory mediators. The results were compared using the Mann-Whitney Rank Sum test with SigmaPlot™ 14 (Systat Software, Inc., San Jose, CA), and significance set at P<0.05.

Supplementary Figure 3 (Fig. S3). Injury characteristics of a subgroup of PALF survivors. Comparison of age and injury characteristics for two patient subgroups of PALF survivors diagnosed with APAP overdose (APAPo, n=3) and non-APAP (n=11). Box plots represent the 25th and 75th percentiles with a line at the median and error bars defining the 10th and 90th percentiles (significance set at *P<0.05, analyzed by Student's t-test or Mann-Whitney Rank Sum test as appropriate).

Supplementary Figure 4 (Fig. S4). Dynamic Network Analysis (DyNA) of inflammatory mediators in mouse hepatocytes. Cell supernatants from freshly isolated mouse hepatocytes from C57BL/6 mice or HC-HMGB1$^{-/-}$ with or without APAP treatment for 1-48h were assayed for 23 inflammatory mediators and DyNA (stringency level 0.85) was as performed as described in *Materials and Methods.* Figure displays the total number of mediator connections across all experimental groups as indicated.

Fig. S1

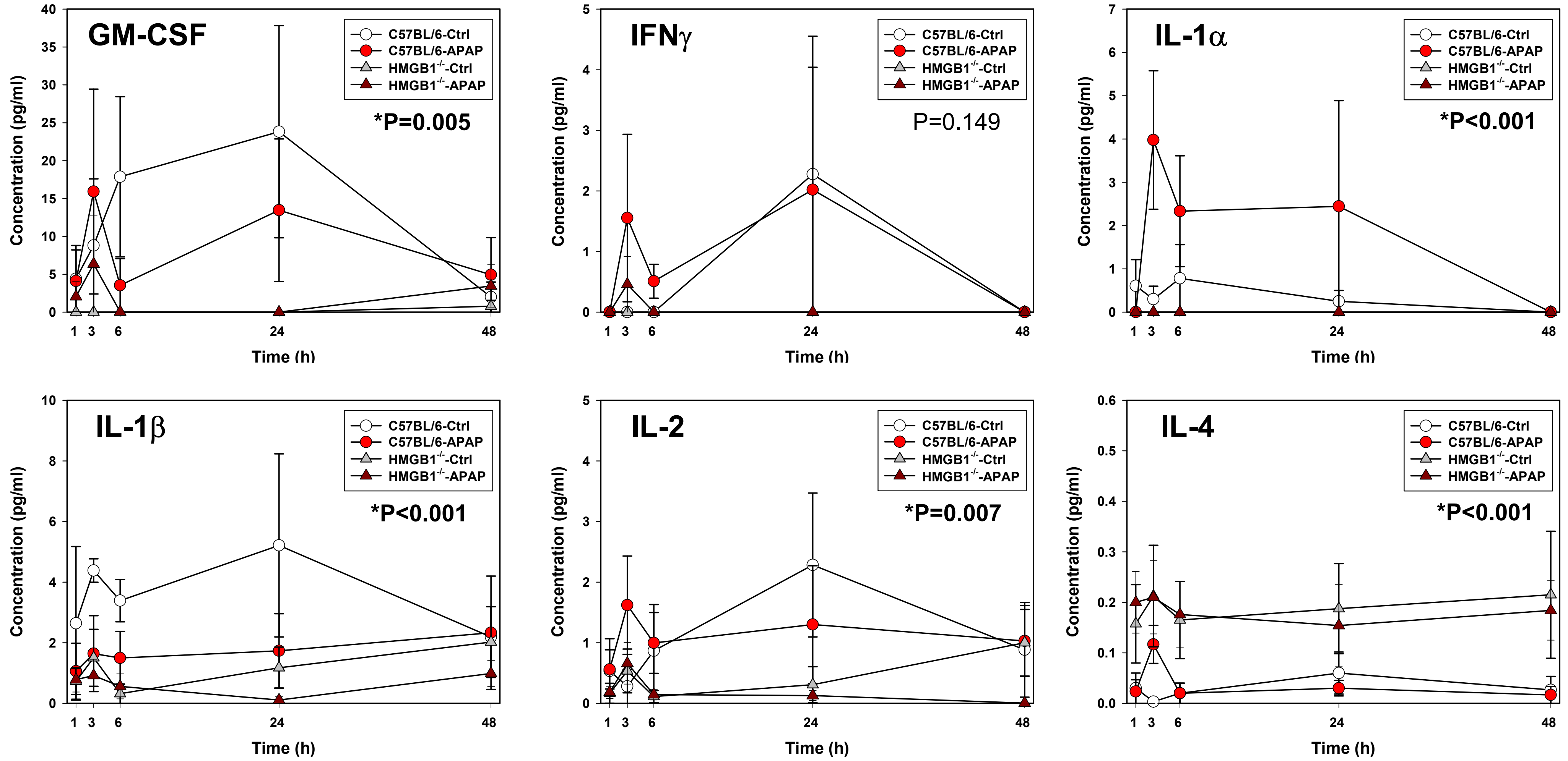
GM-CSF
C57BL/6-Ctrl
C57BL/6-APAP
HMGB1$^{-/-}$-Ctrl
HMGB1$^{-/-}$-APAP
*P=0.005
Concentration (pg/ml)
Time (h)
IFNγ
P=0.149
IL-1α
*P<0.001
IL-1β
*P<0.001
IL-2
*P=0.007
IL-4
*P<0.001

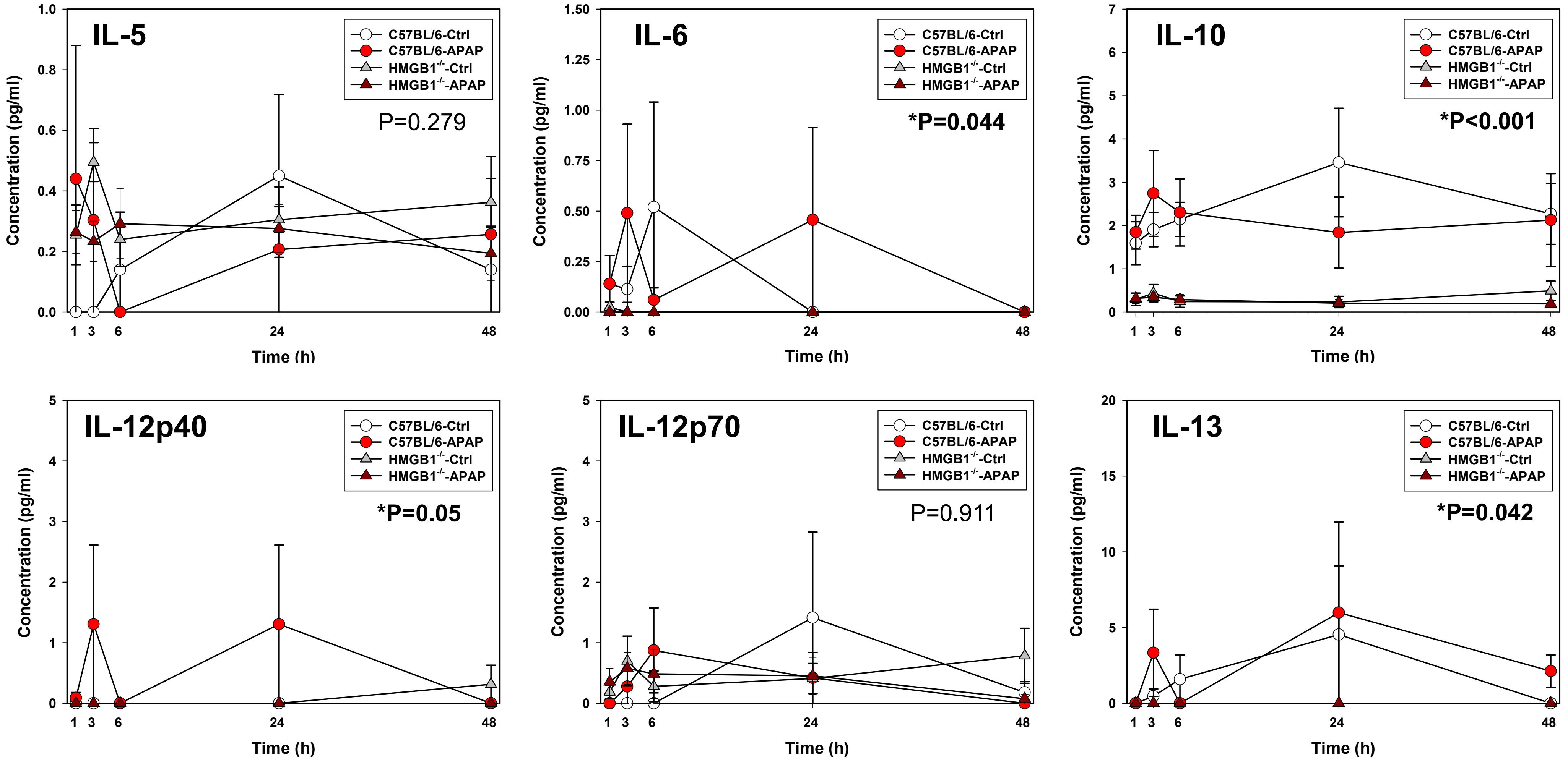
IL-5
P=0.279
IL-6
*P=0.044
IL-10
*P<0.001
IL-12p40
*P=0.05
IL-12p70
P=0.911
IL-13
*P=0.042
C57BL/6-Ctrl
C57BL/6-APAP
HMGB1-/--Ctrl
HMGB1-/--APAP
Concentration (pg/ml)
Time (h)

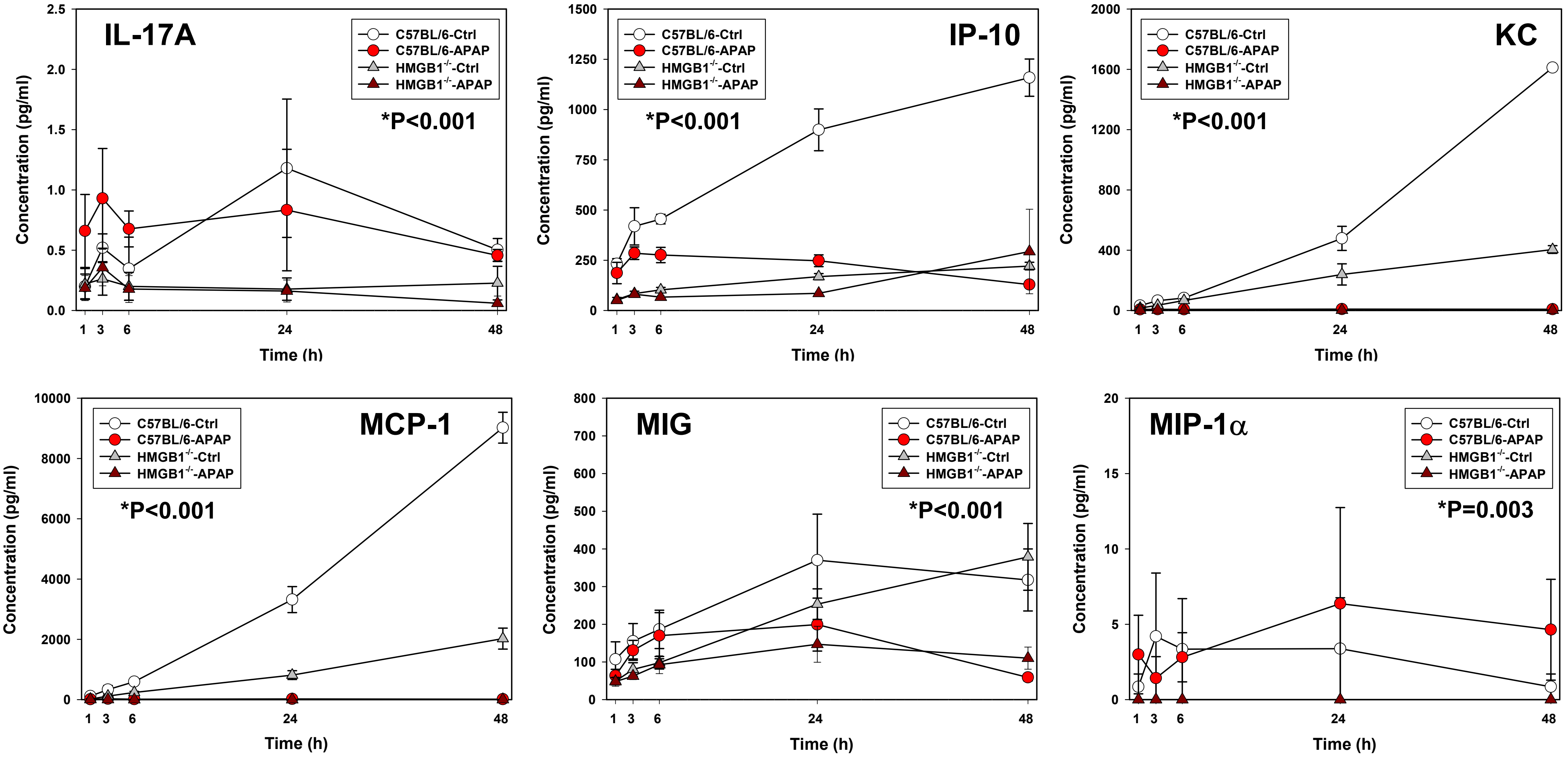
IL-17A
*P<0.001
IP-10
*P<0.001
KC
*P<0.001
MCP-1
*P<0.001
MIG
*P<0.001
MIP-1α
*P=0.003
C57BL/6-Ctrl
C57BL/6-APAP
HMGB1-/--Ctrl
HMGB1-/--APAP
Concentration (pg/ml)
Time (h)
1 3 6 24 48

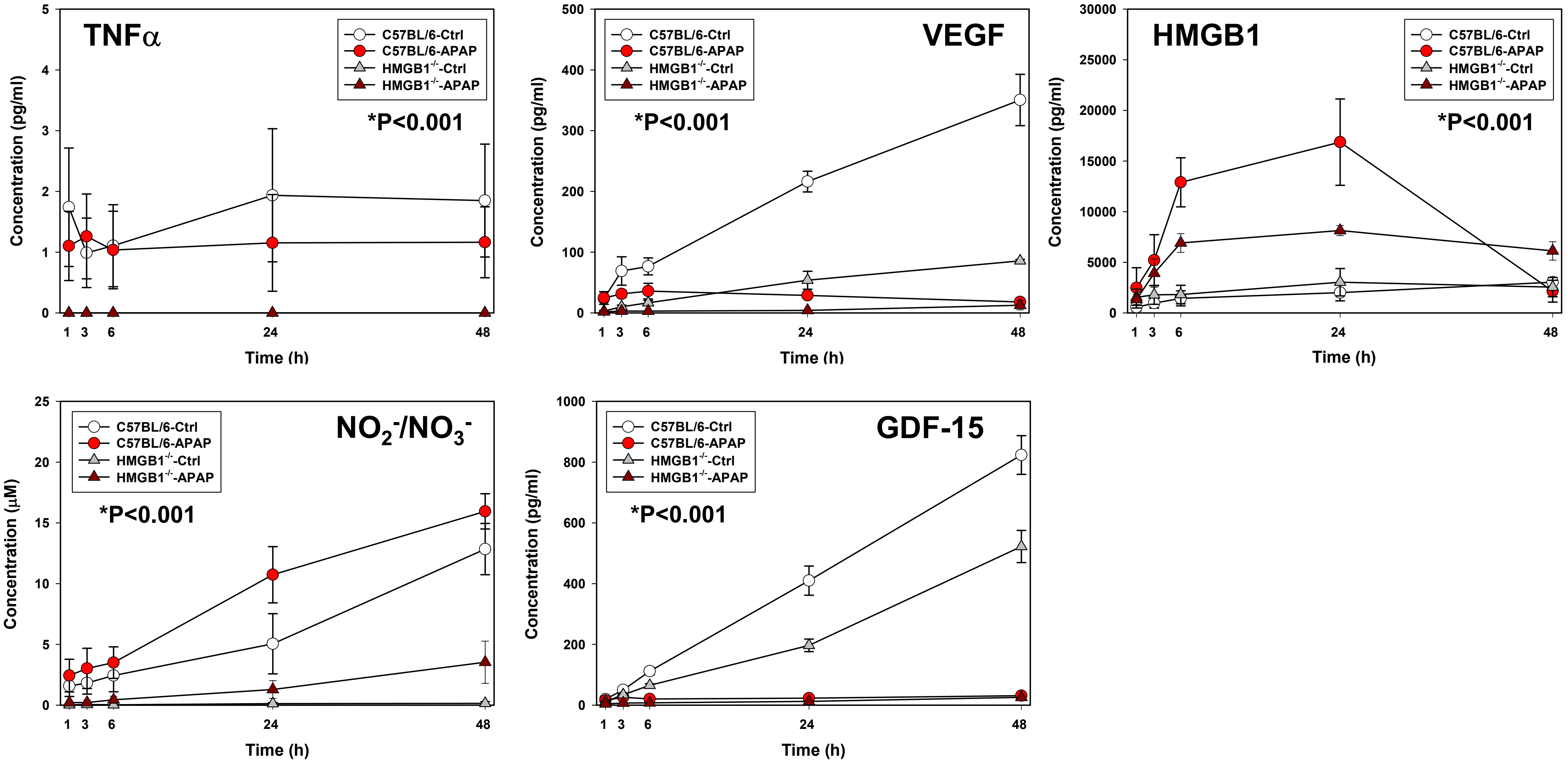
TNFα
VEGF
HMGB1
NO2-/NO3-
GDF-15
*P<0.001
C57BL/6-Ctrl
C57BL/6-APAP
HMGB1-/--Ctrl
HMGB1-/--APAP
Concentration (pg/ml)
Concentration (μM)
Time (h)
1
3
6
24
48

Fig. S2

	Survivors vs. Non-survivors	
	Mediator	P value
1	Eotaxin	0.06
2	GM-CSF	<0.001
3	IFN-α2	0.604
4	IFNγ	0.372
5	IL-1RA	0.801
6	IL-1β	0.011
7	IL-2	0.157
8	sIL-2Rα	0.02
9	IL-4	0.658
10	IL-5	0.231
11	IL-6	<0.001
12	IL-7	0.303
13	IL-8	<0.001
14	IL-10	<0.001
15	IL-12p40	0.035
16	IL-12p70	0.444
17	IL-13	0.001
18	IL-15	<0.001
19	IL-17A	0.599
20	IP-10	0.564
21	MCP-1	<0.001
22	MIG	0.046
23	MIP-1α	0.086
24	MIP-1β	<0.001
25	TNF-α	0.058
26	HMGB1	0.689
27	NO_2^-/NO_3^-	0.239
28	GDF-15	<0.001

Fig. S3

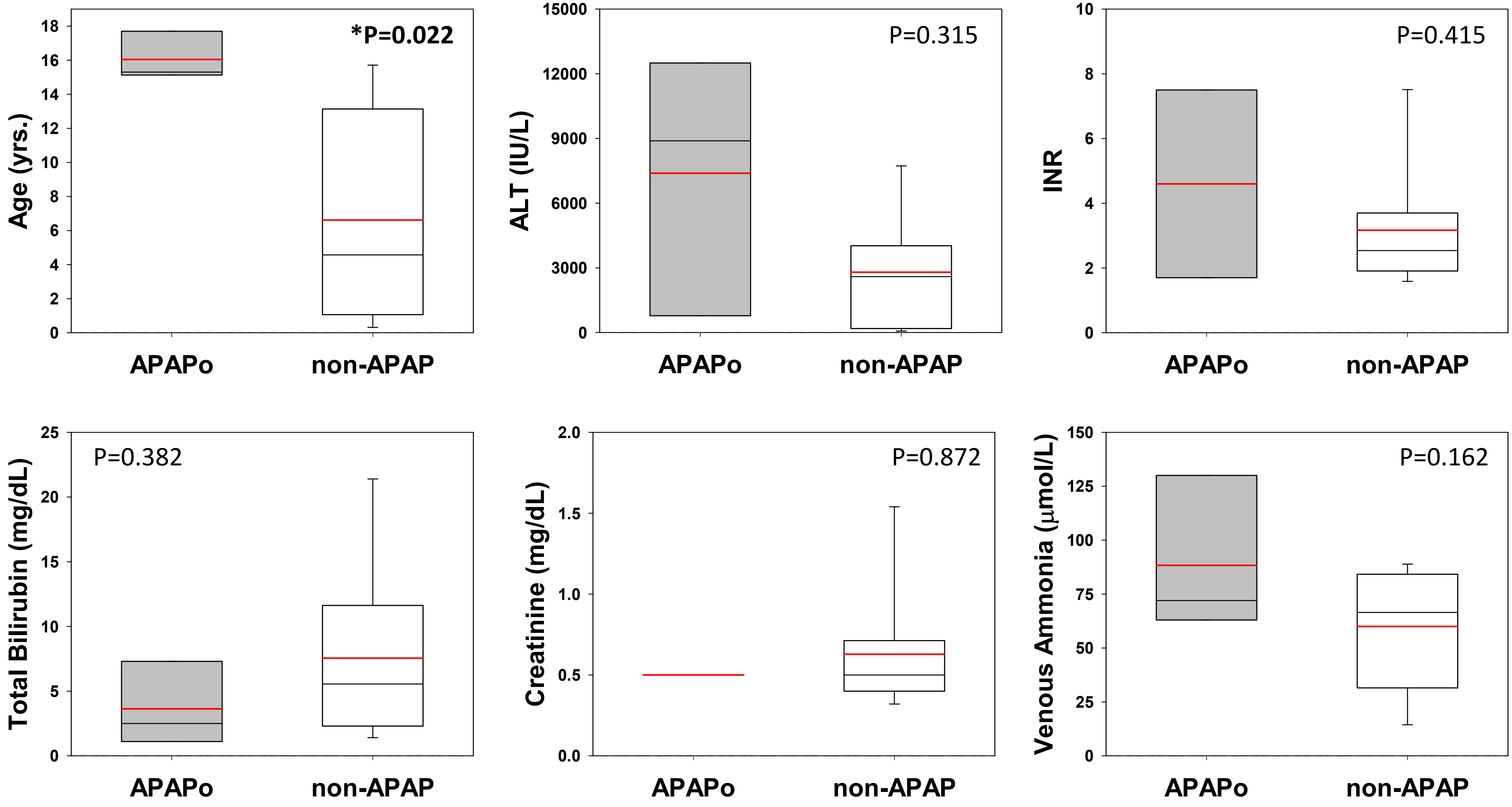

Age (yrs.)
*P=0.022
ALT (IU/L)
P=0.315
INR
P=0.415
Total Bilirubin (mg/dL)
P=0.382
Creatinine (mg/dL)
P=0.872
Venous Ammonia (μmol/L)
P=0.162
APAPo
non-APAP

Fig. S4

C57BL/6 (CTRL)	1h-3h	3h-6h	6h-24h	24h-48h	Sum
Total connections	15	26	33	43	117
Positive connections	10	14	33	43	100
Negative connections	5	12	0	0	17
C57BL/6 (APAP)	1h-3h	3h-6h	6h-24h	24h-48h	Sum
Total connections	27	30	53	43	153
Positive connections	27	30	51	43	151
Negative connections	0	0	2	0	2
HC-HMGB1-/- (CTRL)	1h-3h	3h-6h	6h-24h	24h-48h	Sum
Total connections	12	14	10	17	53
Positive connections	12	14	10	17	53
Negative connections	0	0	0	0	0
HC-HMGB1-/- (APAP)	1h-3h	3h-6h	6h-24h	24h-48h	Sum
Total connections	7	9	8	6	30
Positive connections	6	9	8	6	29
Negative connections	1	0	0	0	1
